# Influence of family resources on secondhand smoking in pregnant women: a cross-sectional study in the border and minority urban areas of Northwest China

**DOI:** 10.1186/s12884-020-03251-w

**Published:** 2020-10-21

**Authors:** Jiangyun Chen, Xinhui Li, Pengqian Fang

**Affiliations:** 1 School of Health Services Management, Southern Medical University, No. 1023-1063 Shatai Road, Baiyun District, Guangzhou, 510515 Guangdong China; 2 School of Medicine, Shihezi University, No.221 Beisi Road, Shihezi, Xinjiang, 832002 Uygur Autonomous Region China; 3 Academy of Health Policy and Management, Huazhong University of Science and Technology (Think tank), No.13 Hangkong Road, Qiaokou District, Wuhan, 430030 Hubei China; 4 School of Medicine and Health Management, Tongji Medical College, Huazhong University of Science and Technology, No.13 Hangkong Road, Qiaokou District, Wuhan, 430030 Hubei China

**Keywords:** Pregnant women, Secondhand smoke, Family resources, Home smoking ban, Religion

## Abstract

**Background:**

Pregnant women’s exposure to secondhand smoke is a very serious health issue in China. The purpose of our research is to identify factors that predict the probability of exposure to secondhand smoke among pregnant women from the perspective of a family-based open system.

**Methods:**

From September 2014 to August 2015, Urumqi City, Shihezi City, and Shawan County-level City were sampled according to population characteristics. A revised structured questionnaire based on family resources was adapted for use in this study. Questionnaires were collected via convenience sampling at the hospitals with the largest number of local antenatal clients. A total of 1249 pregnant women of age 18–51 years were investigated. Descriptive statistics were calculated to characterize the participants and study variables. Binary logistic regression was performed to assess the impact of family resources corresponding variables on the likelihood that participants would be exposed to SHS. Both unadjusted and adjusted odds ratios (OR/AOR) [with 95% confidence intervals (CI)] were reported.

**Results:**

The secondhand smoke exposure rate found in this study was 54.6%. Having good knowledge of the dangers of secondhand smoke had no effect on reducing the prevalence of exposure (*P* > 0.05). Even pregnant women whose husbands who did not use tobacco or never smoked nearby had a risk of exposure to secondhand smoke [adjusted odds ratio (AOR) 1.568, 95% CI 1.205–2.041] when the data were adjusted for age, gravidity, gestational weeks, knowledge of the dangers of secondhand smoke, location, and work status. Home smoking bans were confirmed to be an important protective factor (AOR 1.710, 95% CI 1.549–1.918); however, only one-third (33.5%) of participants reported having a smoking ban at home. Religion (mainly Islam), as a special external family resource, was a protective factor that reduced secondhand smoke exposure in pregnant women (AOR 0.399, 95% CI 0.312–0.510).

**Conclusions:**

The effect of family resources on tobacco control should be considered in the development of effective and enduring strategies for indoor smoking bans and smoking cessation.

## Background

China has the largest number of tobacco consumers in the world, and secondhand smoke (SHS) exposure remains a serious issue. In China, 740 million people are routinely exposed to SHS and 100 hundred deaths related every year [1]. According to the 2018 China Adult Tobacco Survey (CATS), the current prevalence of smoking for females (2.1%) is far lower than that for males (50.5%) [2]. According to the 2010 Global Adult Tobacco Survey (GATS), the percentages of SHS exposure among women aged 15–49 years in China were 65.1% at home and 52.6% at work [3]. Apparently, non-smoking women of reproductive age are one of the main groups exposed to SHS in China.

The harmful effects of SHS have been well documented in studies conducted in China on pregnant women themselves (such as lung cancer, ischaemic heart diseases, stroke, cervical neoplasia, chronic obstructive pulmonary, and insufficient sleep duration) [4–7], and their foetuses or offspring (such low birthweight, congenital malformations, spontaneous abortion, orofacial clefts, and decreased lung function) [8–12].

Currently, the effect of a government anti-smoking policy in China is obvious in administrative office buildings, schools, hospitals, airports, and trains; however, there is no related regulation prohibiting smoking at home. Recent evidence has shown that the use of tobacco in the home environment leads to poor indoor air quality (IAQ) and causes serious SHS, with median PM2.5 concentrations in smoking-permitted homes ten times higher than in smoke-free homes [13]. Thus far, home smoking bans have not been widely adopted by Chinese families (only 20%) [1], and 44.9% of adults reported that someone in their home smoked [2].

A family is a system because it is composed of interrelated parts. The components of the family stem are the individual family members. The environment of the family system is the larger society, the culture in which it is located [14]. General systems theory provides the basis for the conception of the family system [15, 16]. In a family-based open system, pregnant women’s exposure to SHS is the result of a combination of internal and external family resources, which should be explored in terms of local unique social, cultural and religious backgrounds, the smoking behaviours of major family members, etc. It was reported that 71.4% of husbands who smoke did not smoke near their wives during pregnancy [17], and the prevalence of SHS among women decreased from 55.9 to 41.9% after pregnancy [18].

The standardised smoking prevalence varied in different provinces of China, the smoking prevalence in Xinjiang Uyghur Autonomous Region has consistently been below the national average of China, from 15.4% in 2003 to 16.2% in 2013 and 22.7% in 2017 [19, 20]. Lower rates of smoking than the national average of China might due to the majority of the local residents are Muslims. Results of previous studies have suggested that religious activities may have a protective role against cigarette smoking among Muslims [21]. Young adults who attend religious services have lower rates of current and subsequent cigarette smoking [22]. Although lower than the national level, the smoking rate in Xinjiang is still on the rise. The risks of secondhand smoking prevalence may be affected by these trends, and the issue of passive smoking should cause more attention.

There are few investigations on such special region and the autonomous province multi-ethnic people’s passive smoking status and analyse the influencing factors. This study is the first to our knowledge to focus on secondhand smoke among pregnant women from the perspective of a family-based open system in an ethnically diverse area of China. The objective of this study of non-smoking pregnant women in the Xinjiang Uyghur Autonomous Region was to identify SHS exposure-associated factors considering family resources, including social, cultural, religious, economic and medical aspects [23].

## Subjects and methods

### Sample strategy

From September 2014 to August 2015, a hospital-based cross-sectional study was conducted in Urumqi City, Shihezi City, and Shawan County-level City in the Xinjiang Uygur Autonomous Region. These cities were selected using typical sampling. In terms of city size (small, medium and large cities, which have populations of > 0.2 ~ 0.5 million, > 0.5 ~ 1 million, > 1 ~ 5 million, respectively), Urumqi City is the only large city in Xinjiang Uygur Autonomous Region and is one of the cities with the highest proportion of Uyghurs (30.83%), Shihezi City is a medium city and has the highest proportion of Han (94.84%), and Shawan County-level City is a small city and has the highest proportion of Kazakhs (52.49%). Questionnaires were collected via convenience sampling at the hospitals with the largest number of antenatal clients (Xinjiang Autonomous Region People’s Hospital, Shihezi City People’s Hospital, and Shawan County Maternal and Child Health Hospital). The inclusion criteria for this study were as follows: (a) an intrauterine pregnancy, (b) the provision of informed consent, (c) local residency, defined as having lived in the area for a cumulative total of 6 months in the last year. The exclusion criteria were (a) mental incompetence and (b) inability to complete the survey for other reasons.

As most pregnant women had fetal heart monitoring every week, medical personnel helped to recruit each eligible target population after finished a prenatal examination. Then eligible participants were informed of the study purpose by investigator and requested to participate in the investigation. After obtaining approval, investigations were conducted in the antenatal clinics of the hospitals. The questionnaire was completed by the participants themselves. Investigators participated in the survey throughout the process and recovered all questionnaires. Uighur/Kazakh nurses were present to help minority participants who had difficulty reading Chinese.

### Sample size

To ensure that our sample of non-smoking pregnant women was of sufficient size, we calculated the power using the maternal passive smoking rate during pregnancy (*P)* in China (which ranged from 26.6 to 45.3% in recent years) [23–25]. Specifically, using the survey sample size calculation formula (*n* = *t*_*α*_^*2*^*PQ/d*^*2*^) with a *P* = 26.6%, an allowable error (*d*) of 0.1*P* and a predetermined α of 0.05, the required sample size of pregnant women was 1104. Of 1395 eligible pregnant women, 1327 (95.1%) consented to participate. A total of 1327 questionnaires were distributed and recovered, with a response rate of 100%. Seventy-eight questionnaires were removed from the study because of incomplete answers or illogical errors. A total of 1249 usable questionnaires were collected for an efficiency of 94.1%.

### Questionnaire

Family resources are defined as the material and spiritual support needed to maintain basic family functions and respond to stressful events and crisis situations. The adequacy of family resources is directly related to the ability of family members to adapt to stress and crisis. Family resources can be divided into domestic resources and extra-family resources (Table 1), which include social, cultural, religious, economic and medical aspects [23, 26]. According to family resources and corresponding variables, a revised structured questionnaire with a Kaiser-Meyer-Olkin value of 0.816 was adapted for use in this study (Supplementary questionnaire file 1).

**Table 1:** Family resources and corresponding variables

Dimensions	Variables
Internal family resources	
Financial support	Annual income
Advocacy/Maintenance	Home smoking ban, Husband tobacco use, Household tobacco use
Medical management	Regular prenatal checkup
Love support	Marital relationships
Information and knowledge	Age, Gravidity times, Gestational weeks, Knowledge of secondhand smoke harm
Structural support	Residence or facility changes
External family resources	
Social resources	Community tobacco control education
Cultural resources	Nationality
Religious resources	Religion
Economic resources	Insurance
Educational resources	Education
Environmental resources	Location, Work status
Medical resources	Have medical institutions in the living community

The questionnaire consisted of two parts: (a) The first part focused on demographic characteristics such as age, location, nationality, religion, gravidity, gestational weeks, education, work status and income, whether regular prenatal checkups took place, and residence or facility changes; (b) The second part focused on passive smoking status (exposure to environmental tobacco smoke at least 1 day per week, regardless of exposure time) and related characteristics, such as home smoking bans, husband’s tobacco use, household tobacco use, community tobacco control education, etc.

In addition, a marital relationships scale and a secondhand smoke harm knowledge scale were provided. After reviewing and revising the marital satisfaction scale [27], we measured marital relationships with 20 questions about self-perceptions of the partner and marital life that were answered via a four-point Likert scale. The Cronbach’s alpha of this scale was 0.878. The score is between 20 and 80 points. A higher score means that respondents tend to be satisfied with the marriage. Six items answered using a four-point Likert were used to measure the knowledge level of secondhand smoke harm by referencing SHS health hazards from the website of Chinese Centre for Control and Prevention (CDC) [28], and the Cronbach’s alpha was 0.826. The score is between 6 and 24 points. A higher score indicates better knowledge of the harms of secondhand smoke.

### Definition of SHS

According to the definition in the Global Adult Tobacco Survey 2010, secondhand smoke exposure (SHS) is defined as the exposure of a non-smoker to environmental tobacco smoke at least 1 day per week, and the rate of secondhand smoke exposure is the percentage of non-smokers who are exposed to secondhand smoke [29].

### Data analysis

The Epidata 3.1 software was used to create a database and enter data. SPSS statistical software version 20.0 for Windows was used for all data analyses. Descriptive statistics were calculated to characterize the participants and study variables. The chi-square test was used to compare the distribution of the SHS exposure rate between different social demographic characteristics groups. The Spearman correlation test was used to assess the correlations among variables. We used self-rated SHS exposure as the binary dependent variable. Ten independent variables were significant at the<0.10 level (home smoking bans, husband’s tobacco use, household tobacco use, age, gravidity, gestational weeks, knowledge of the dangers of secondhand smoke, religion, location, work status) and were selected for inclusion in the model. Using a criterion of a *p*-value under 0.05, binary logistic regression was performed to assess the impact of these variables on the likelihood that participants would be exposed to SHS. Both unadjusted and adjusted odds ratios (OR/AOR) [with 95% confidence intervals (CI)] were reported.

## Results

### Characteristics of participants

Among the sample of 1249, 32.1% were from the Urumqi, 23.8% from Shihezi and 12.3% from Shawan. The mean (SD) reported age was 29.1 years (4.6). One-half (52.0%) of the participants were Han, and one-quarter (25.5%) of them were Uygur, followed by Kazak (12.9%). Less than half (46.0%) of the participants reported having religious beliefs, mainly Islam (*n* = 556, 96.9%). Approximately half (54.2%) of the participants were primigravida, and most (92.2%) of the participants were more than 12 weeks pregnant. The majority (66.5%) of the participants reported having completed high school, and three-quarter (75.5%) of the participants reported working during their pregnancy. More than half (53.5%) of the participants reported a household per capita annual income of USD 5421 ~ 12,649. Only a few (3.4%) of the participants had no health insurance. Almost all (99.6%) the participants had medical institutions in the community where they lived. The marital relationship scale scores were (60.6 ± 5.7), and the secondhand smoke harm knowledge scale score were (21.7 ± 2.7), which were both in the upper middle level.

### SHS prevalence rate and distribution

The overall rate of secondhand smoke exposure among the pregnant women in this investigation was 54.6% (682/1249). Participants having home smoking ban and participants with religious beliefs reported lower rate of SHS prevalence for 47.6 and 43.3%, respectively. The prevalence of SHS was higher among participants whose husbands smoked. The SHS prevalence rate of the husband smoking nearby was as high as 92.8%, and even if the husband was not smoking nearby, the SHS prevalence rate also reached 57.2%.

The chi-square test was used to compare the distributions of SHS exposure rate according to family resource characteristics. The results showed statistically significant differences in the distribution of the secondhand smoke exposure rate for the following variables: home smoking bans, husband’s tobacco use, age, gravidity, gestational weeks, knowledge of the harms of secondhand smoke, household tobacco use, nationality, religion, location, and work status (*P* < 0.1) (Table 2, Table 3).

**Table 2:** Comparison of secondhand smoke exposure rate among different internal family resources (*n* = 1249)

Internal family resources Dimensions	Variables	Overall *N* = 1249(%), mean (SD)	Secondhand smokers *N* = 682(%)	Rate of secondhand smoke exposure (%)	χ^2^	*P* value
Financial support	Annual income (USD)				0.602	0.740
	< 5421	226 (18.1)	122 (17.9)	54.0		
	5421 ~ 12,649	668 (53.5)	360 (52.8)	53.9		
	> 12,649	355 (28.4)	200 (29.3)	56.3		
Advocacy/Maintenance	Home smoking ban				12.405	< 0.001
	Yes	418 (33.5)	199 (29.2)	47.6		
	No	831 (66.5)	483 (70.8)	58.1		
	Husband tobacco use				117.199	< 0.001
	No tobacco use	702 (56.2)	315 (46.2)	44.9		
	Smoke but not nearby	395 (31.6)	226 (33.1)	57.2		
	Smoke nearby	152 (12.2)	141 (20.7)	92.8		
	Household tobacco use (except husband)				27.252	< 0.001
	No tobacco use	791 (63.3)	403 (59.0)	50.9		
	Smoke but not nearby	348 (27.9)	194 (28.5)	55.9		
	Smoke nearby	110 (8.8)	85 (12.5)	77.3		
Medical management	Regular prenatal checkup				0.487	0.485
	Yes	1202 (96.2)	654 (95.9)	54.4		
	No	47 (3.8)	28 (4.1)	59.6		
Love support	Marital relationships				1.751	0.186
	≤61	633 (50.7)	334 (53.2)	52.8		
	> 61	616 (49.3)	348 (46.8)	56.5		
Information and knowledge	Age	29.1 ± 4.6			10.461	0.001
	≥29	642 (51.4)	379 (55.6)	59.0		
	< 29	607 (48.6)	303 (44.4)	49.9		
	Gravidity times				3.059	0.080
	1	677 (54.2)	385 (56.5)	56.9		
	≥2	572 (45.8)	297 (43.5)	51.9		
	Gestational weeks				5.944	0.051
	≤12	98 (7.8)	53 (7.8)	54.1		
	13 ~ 28	432 (34.6)	256 (37.5)	59.3		
	> 28	719 (57.6)	373 (54.7)	51.9		
	Knowledge of secondhand smoke harm				3.874	0.053
	≤22	591 (47.3)	340 (50.1)	57.5		
	> 22	658 (52.7)	342 (49.9)	52.0		
Structural support	Residence or facility changes				0.809	0.368
	Yes	58 (4.6)	35 (5.1)	60.3		
	No	1191 (95.4)	647 (94.9)	54.3

**Table 3:** Comparison of secondhand smoke exposure rate among different external family resources (*n* = 1249)

External family resources Dimensions	Variables	Overall *N* = 1249(%), mean (SD)	Secondhand smokers *N* = 682(%)	Rate of secondhand smoke exposure (%)	χ^2^	*P* value
Social resources	Community tobacco control education				0.014	0.906
	Yes	868 (69.5)	473 (69.4)	54.5		
	No	381 (30.5)	209 (30.6)	54.9		
Cultural resources	Nationality				54.080	< 0.001
	Han	650 (52.0)	417 (61.1)	64.2		
	Uygur	319 (25.5)	137 (20.1)	42.9		
	Kazak	161 (12.9)	70 (10.3)	43.5		
	Hui	103 (8.3)	47 (6.9)	45.6		
	others	16 (1.3)	11 (1.6)	68.8		
Religious resources	Religion				53.978	< 0.001
	Yes	574 (46.0)	249 (36.5)	43.3		
	No	675 (54.0)	433 (63.5)	64.1		
Economic resources	Insurance				0.225	0.636
	Yes	1206 (96.6)	657 (96.3)	54.5		
	No	43 (3.4)	25 (3.7)	58.1		
Educational resources	Education				2.370	0.124
	High school or less	419 (33.5)	216 (31.7)	51.6		
	Above high school	830 (66.5)	466 (68.3)	56.1		
Environmental resources	Location				13.302	0.001
	Urumqi	622 (49.8)	321 (47.1)	51.6		
	Shihezi	382 (30.6)	238 (34.9)	62.3		
	Shawan	245 (19.6)	123 (18.0)	50.2		
	Work during pregnancy				4.196	0.041
	Yes	946 (75.7)	532 (78.0)	56.2		
	No	303 (24.3)	150 (22.0)	49.5		

### Influencing factors of SHS

To investigate the correlations among the eleven variables identified in the bivariate analysis, Spearman’s rho correlation coefficient was calculated (Table 4). The results showed that there might be a positive correlation between nationality and religion, with *r* = 0.806 (*P* < 0.001). In this study, there was an overlap in religions among different nationalities, and Islam is also believed by Han, Uyghurs, Kazaks and others. To avoid the interference of confounding factors, when there was a collinearity between nationality and religion, religion was selected for inclusion in the model development.

**Table 4:** Correlation coefficient calculated between variables (*n* = 1249)

Correlation Coefficient	Home smoking ban	Husband tobacco use	Household tobacco use (except husband)	age	Gravidity times	Gestational weeks	Knowledge of secondhand smoke harm	nationality	religion	location	Work status
Home smoking ban	1.000										
Husband tobacco use	0.170^**^	1.000									
Household tobacco use (except husband)	−0.062^*^	0.020	1.000								
age	0.046	−0.020	0.083^**^	1.000							
Gravidity times	− 0.097^**^	0.042	0.016	−0.360^**^	1.000						
Gestational weeks	0.047	0.007	0.004	0.092^**^	−0.080^**^	1.000					
Knowledge of secondhand smoke harm	0.210^**^	−0.092^**^	− 0.017	0.087^**^	0.127^**^	−0.018	1.000				
nationality	0.028	−0.053	− 0.052	0.079^**^	− 0.058^*^	0.085^**^	0.057^*^	1.000			
religion	−0.016	− 0.076^**^	− 0.037	0.068^*^	0.003	0.065^*^	0.121^**^	0.806^**^	1.000		
location	0.173^**^	−0.046	−0.066^*^	−0.086^**^	− 0.166^**^	0.013	− 0.215^**^	0.133^**^	− 0.107^**^	1.000	
Work status	−0.074^**^	−0.009	0.029	0.054	0.001	−0.037	0.051	0.074^**^	0.058^*^	−0.020	1.000

**. Correlation is significant at the 0.01 level (2-tailed)

*. Correlation is significant at the 0.05 level (2-tailed)

Binary logistic regression was performed to assess the impact of ten significant independent variables on the likelihood that participants would be exposed to SHS. The full model was statistically significant, χ^2^ = 246.32, *P* < 0.001. Only five of the variables made a unique statistically significant contribution to the model (Table 5). The strongest predictor of exposure to SHS was having a husband who smoked nearby (versus a husband who did not use tobacco), with an AOR of 17.438, 95% CI (9.139–33.276), followed by having a household member who smokes nearby (versus no household who use tobacco) [AOR 2.232, 95% CI (1.351–3.687)], allowing smoking allowed in the home [AOR 1.710, 95% CI (1.549–1.918)], and having a husband who smokes, but not nearby (versus a husband who did not use tobacco) [AOR 1.568, 95% CI (1.205–2.041)]. Women who had a religious affiliation (versus those without a religion) [AOR 0.399, 95% CI (0.310–0.510)] and a gravidity of 2 or more (versus the first gravidity) [AOR 0.731, 95% CI (0.570–0.937)] had a lower likelihood of SHS.

**Table 5:** Binary logistic regression predicting likelihood of pregnant women exposed to SHS (*n* = 1249)

	Unadjusted ^a^		*p* value	Adjusted ^b^		*p* value
	OR	95% CI		OR	95% CI	
Religion^c^	0.428	(0.341*–*0.358)	< 0.001	0.399	(0.312*–*0.510)	< 0.001
Gravidity times^d^	0.819	(0.655*–*1.024)	0.080	0.731	(0.570*–*0.937)	0.013
Smoking allowed at home^e^	1.655	(1.517*–*1.829)	< 0.001	1.710	(1.549*–*1.918)	0.001
Husband smoke but not nearby (no tobacco use)^f^	1.643	(1.281*–*2.107)	< 0.001	1.568	(1.205*–*2.041)	0.001
Husband smoke nearby (no tobacco use)^f^	15.748	(8.376*–*29.608)	< 0.001	17.438	(9.139*–*33.276)	< 0.001
Household tobacco user (except husband) smoke nearby (no tobacco use)^f^	3.040	(1.916*–*4.823)	< 0.001	2.232	(1.351*–*3.687)	0.002

Adjusted with age, gravidity times, gestational weeks, knowledge of secondhand smoke harm, religion, location, work status

^a^Significance *p* < 0.10

^b^Significance *p* < 0.05

^c^Reference category: Have a religion (versus non-religion)

^d^Reference category: Gravidity times of 2 or more (versus the first gravidity)

^e^Reference category: ‘Allowed’ (versus ‘Never allowed’ and ‘Not allowed, as a general rule’)

^f^Definition: ‘nearby’ is in an extent of 5 m

## Discussion

The overall national prevalence of SHS among pregnant women in China has not yet been determined. A previous study showed that in terms of exposure to SHS at home amongst women aged 15–49 years, China had one of the highest rates (65.1%) of the 14 low- and middle-income countries included (Bangladesh, Brazil, China, Egypt, India, Mexico, Philippines, Poland, Russian Federation, Thailand, Turkey, Ukraine, Uruguay, and Vietnam) [30]. The majority of pregnant women fall into this age group; presumably, SHS exposure among pregnant women is not lower in China than in other countries. Compared to other domestic regional cross-sectional studies, the SHS prevalence rate found in this study (northwest China, 54.6%) was the same as that in southwest China (58.5%) and was higher than that in the central region of China (34.26%) and in eastern China (45.3%) [25, 31, 32].

An analysis of the probability of exposure to SHS shows that women who had a gravidity of 2 or more had lower chances of SHS exposure [AOR 0.731, 95% CI (0.570, 0.937)] compared with primigravid women. Women in their first pregnancy may lack pregnancy-related health knowledge (e.g., lack of awareness and effective experience regarding secondhand smoke exposure prevention). In addition, under China’s one-child policy, which was in place during the investigation, women with more than one pregnancy may have experienced miscarriage. Therefore, they may be very concerned about the risk factors for pregnancy health and may have received more attention and understanding from their family systems, which may have contributed to the reduced exposure to SHS.

Similar to previous results [24], our results found that the primary source of SHS for pregnant women was husbands who smoke, followed by other household members who smoked. The difference is that we also found effects for various smoking behaviours of the husbands. The majority of the smoking husbands (76.0%, 348/458) tried to avoid smoking near their pregnant wives in consideration of maternal and foetal health, but the risk of SHS exposure still existed [AOR 1.568, 95% CI (1.205–2.041)]. Despite the husbands’ indoor smoking behaviours, environmental tobacco smoke from the spontaneous combustion of cigarettes can also cause SHS exposure for pregnant women, along with the potential risk of third-hand smoke. These husbands are to be commended for their adjustment of their smoking behaviour. It is believed that correct guidance regarding smoking avoidance and smoking cessation can optimize the willingness of smoking husbands to make changes in the family system.

Interestingly, this study found that good knowledge of the harms of SHS had no effect on reducing the prevalence of SHS. Smoking is socially acceptable in China; 47.0% of families reported sharing cigarettes with others, and 14.17% of families reported giving cigarettes as a gift [33]. Pregnant Chinese women have always engaged in preventive behaviours by avoiding indoor SHS exposure when strangers smoke, but this behaviour was not obvious when acquaintances smoke because of concerns about disrupting family harmony and interpersonal relationships [34]. This result shows that pregnant women’s SHS exposure is affected by the combined effects of family resources, and it was difficult to avoid SHS without the support of cultural and social resources, even with sufficient information and knowledge. The latest evidence suggests that tobacco exposure during the few months before conception, even with cessation in the first trimester, may also pose a risk for fetal malformation [35]. Therefore, it is necessary to change public smoking customs in China and involve people in both preconception and pregnancy health education.

Pregnant women who live in homes where smoking is allowed have a higher SHS exposure rate than those who do not [AOR 1.710, 95% CI (1.549–1.918)]. Home smoking bans were confirmed to be an important protective factor in this study; however, it has been noted that China has the worst rates of smoking control in the home among the International Tobacco Control Policy Evaluation Project (ITC) countries, and only 20% of households do not allow smoking in the home [1]. In this study, one-third (33.5%) of the participants reported having a smoking ban indoors; although this is higher than the rate reported by the ITC, it is still very low. China has not yet enacted legislation prohibiting smoking at home. Moreover, because women spend more time at home after pregnancy, creating a smoke-free home environment becomes seriously important. The implementation of indoor smoking bans and restrictions on tobacco advertising are the main evidence-based policy recommendations by the WHO FCTC; these strategies have proven to be among the most successful tobacco control policies and have significant policy and programmatic implications [36, 37]. In addition, the literature indicates that the involvement of women in family decision-making is a factor in the adoption of smoke-free homes [38, 39]. Home smoking bans were shown to be maintained and supported by family members out of concern for pregnant women’s health. Therefore, in addition to the ongoing implementation of national tobacco control policies, encouraging and promoting the maintenance and prioritization of maternal health throughout the family is a necessary condition for the creation of smoke-free homes.

We also found some new, interesting results in this study. Religion was a protective factor that reduced the SHS exposure of pregnant women [AOR 0.399, 95% CI (0.312–0.510)]. The SHS prevalence rate reported by pregnant women who had a religious affiliation (96.9% were Islam) was much lower than those who had no religion. Xinjiang Uygur Autonomous Region is a typical multi-nationality and multi-religion region. Islam, as minority religion, is followed by the Uygur, Kazak, Hui and other more than 10 nationality groups [40]. According to *the Qur’an*, Muhammad once said *“Verily spendthrifts are brothers of the Evil Ones; and the Evil One is to his Lord (himself) ungrateful”*, and *“Everything that is intoxicating (narcotic, addictive) is prohibited”* [41, 42]. According to Islam tenets, smoking is an extravagant behaviour that produces SHS and harm the health of oneself and others; for Muslims, it is a sin that should not be practiced. In this study, the pregnant women held religious beliefs were primarily adherents of Islam, who are opposed to smoking and are unlikely to have contact with smokers [43]. Moreover, the mosque/mullahs preach the harms of smoking/SHS and restrict tobacco use in many rituals of Muslim life [44], and religious involvement is inversely related to current smoking [45]. Adequate religious resources might objectively contribute to reducing the SHS prevalence rate of pregnant women in the Muslim community.

### Strengths and limitations

This is the first published study from the perspective of a family-based open system to focus on SHS exposure status and its influencing factors among urban multi-nationality pregnant women in the border and minority areas in China. The results of this study may aid in the development of effective and enduring strategies to protect the health of pregnant women and foetuses.

There were several limitations of this study. First, the outcome variables relied on self-report, which can be susceptible to non-sampling errors. However, the self-report methodology has been widely used in tobacco prevalence research and has been proven to be a valid accurate estimate when compared with biomarker findings [37, 46, 47]. Second, as gestational age increases, pregnant women usually undergo a higher frequency of foetal examinations (e.g., pregnant women in late pregnancy are required to undergo foetal heart monitoring once or twice a week). Consequently, women in middle and late pregnancy are more likely to be examined than those in early pregnancy, which may have led to sampling errors. The sample may not be a good indicator of SHS exposure in early pregnancy. Additionally, the study did not revise the Uighur and Kazak Language questionnaire for minority pregnant women, and data were collected with the help of trained minority nationality bilingual translators. In future studies, a questionnaire suitable for minority pregnant women can be created.

## Conclusions

To sum up, this study found that the SHS prevalence rate among pregnant women was at the upper level compared to other domestic regional cross-sectional studies. Having good knowledge of the harms of SHS was not enough to reduce exposure to SHS. Although smoking husbands consciously made efforts to protect pregnant women from exposure to their secondhand smoke, the risk still existed. Therefore, our results suggest that it is very important to help smoking husbands to reduce tobacco use or to be able to correctly and effectively avoid smoking nearby. Home smoking bans were an important protective factor against SHS exposure, but such bans were in effect for only one-third of the participants. Religion, as a special resource, was an important factor in reducing SHS exposure in pregnant women who had an instinctive response (e.g., a tendency to reduce their contact with smokers) and lived in a Muslim community characterized by less SHS. The effect of religious tenets on tobacco control should be considered in the development of effective and enduring strategies for indoor smoking bans and smoking cessation in minority communities. The effect of Islam’s religion tenets regarding tobacco control in this study provides perspective for further research in this field, which is especially necessary within the context of this current era of religious awakening in China.

## Supplementary information

**Additional file 1.** Supplementary questionnaire file 1.

## Data Availability

The datasets used and/or analyzed during the current study are available from the corresponding author on reasonable request.

## Abbreviations

SHS: Secondhand Smoke
IAQ: Indoor Air Quality
PM2.5: Particles with a diameter less than or equal to 2.5 Micrometers
WHO FCTC: The World Health Organization Framework Convention on Tobacco Control
USD: United States Dollar
ITC: The International Tobacco Control Policy Evaluation Project
