# Supplementary material for: Influence of family resources on secondhand smoking in pregnant women: a cross-sectional study in the border and minority urban areas of Northwest China
Source: BMC Pregnancy Childbirth. 2020 Oct 21;20:642. doi: 10.1186/s12884-020-03251-w (PMC7579793; doi:10.1186/s12884-020-03251-w)
Supplement: Supplementary file 1 — Additional file 1. Supplementary questionnaire file 1. [file 12884_2020_3251_MOESM1_ESM.docx]

**TRANSLATED QUESTIONNAIRE**

**Questionnaire of Secondhand Smoke Status among pregnant women**

Number: ……………

**Informed Consent**

Dear expectant moms：

Hello! This questionnaire survey focuses on the status quo of secondhand smoke for pregnant women. The study purpose is to identify factors that predict the probability of exposure to secondhand smoke among pregnant women, and to provide a scientific basis for improving the living environment of pregnant women and promote the health of pregnant women.

It will take about 15 minutes to complete the questionnaire. We will strictly abide by the principle of confidentiality and will not disclose any personal information about you. The data obtained will be used for scientific research purposes only. Your true answer will be of great help to our research. Sincerely thank you for your participation and support!

Research Team

**Note:** The concept of “passive smoking”/“secondhand smoke” in this survey is defined as “non-smoker exposed to environmental tobacco smoke at least 1 day per week, which comes from the smoke emitted by smokers or the spontaneous combustion of cigarettes”.

Name………………………… Tel……………………………

Address………………………

Date…………………………… Signed………………………

**A Personal information**

A1 Age: ……………

A2 Location: ……………

1 Urumqi 2 Shihezi 3 Shawan

A3 How long do you live in current residence? ……………

1 *<*1year 2 ≥1year，years you have resided:……………

A4 Nationality: ……………

1 Han 2 Uygur 3 Kazak 4 Hui 5 other

A5 Religion: ……………

1 Islam 2 Buddhist 3 Taoist 4 Confucian 5 other

A6 Gravidity times: ……………

A7 Gestational weeks: ……………

A8 Education: ……………

1 primary school 2 middle school 3 senior high school Taoist

4 undergraduate 5 postgraduate

A9 Are you still working by now? ……………

1 yes 2 no

A10 Annual income of family? (Yuan) ……………

**B** **Passive smoking related information**

B1 Smoke allowed in your home?

1 yes 2 not allowed, as a general rule 3 no, never

B2 What is the number of cigarettes used by your husband in his lifetime? ……………

1 no (skip to B7) 2 yes, and less than 100 (skip to B7) 3 yes, and more than 100

B3 Dose your husband smoke now?

1 yes, and every day (skip to B5) 2 yes, but not every day (skip to B5)

3 no, quit smoke

B4 How long has your husband gave up smoking? ……………

1 *<*1 month 2 ≥ 1 ~＜6 months 3 6 months ~ ＜1 year

4 ≥ 1 ~＜5 years 5 ≥ 5 ~＜10 years 6 ≥10 years

B5 What is the average number of cigarettes your husband is smoking per day now? ……………

B6 What is the average number of cigarettes your husband is smoking per day nearby you? (In an extent of 5 meters) ……………

B7 Do you have a member of household who smoke? (Except husband) ……………

1 yes 2 no (skip to B10)

B8 What is the average number of cigarettes household member is smoking per day now? ……………

B9 What is the average number of cigarettes household member is smoking per day nearby you? (In an extent of 5 meters) …………

B10 In the past 1 week, do you expose to environmental tobacco smoke at least 15min in 1 day?

1 yes 2 no (complete the questionnaire)

B11 In the past 1 week, how long do you expose to environmental tobacco smoke in total? ……………

1 ＜1 hour 2 ≥1 ~＜7 hours 3 ≥7 ~＜14 hours

4 ＜14 hours 5 ≥14 hours

**C Your views on your spouse and your relationship.**

Please mark “√” on the number that match your opinion. These issues are purely personal and there is no right or wrong.

Strongly Generally Generally Strongly

agree agree disagree disagree

C1 I worry a lot about my marriage. 1 2 3 4

C2 If I could start over again, I would marry someone 1 2 3 4

other than my present spouse.

C3 My marriage is too confining to suit me. 1 2 3 4

C4 I feel that I am “in a rut” in my marriage. 1 2 3 4

C5 I know where I stand with my spouse. 1 2 3 4

C6 I feel competent and fully able to handle my marriage. 1 2 3 4

C7 I get discouraged trying to make my marriage work out. 1 2 3 4

C8 I consider my marital situation to be as pleasant as it should be. 1 2 3 4

C9 I think my marriage gets more difficult for me each year. 1 2 3 4

C10 My spouse gets me badly flustered and jittery. 1 2 3 4

C1l I have made a success of my marriage so far. 1 2 3 4

C12 The future of my marriage looks promising to me. 1 2 3 4

C13 I am really interested in my spouse. 1 2 3 4

C14 I get along well with my spouse. 1 2 3 4

C15 My marriage helps me towards goals I have set up for myself. 1 2 3 4

C16 My spouse to make helpful improvements in our relationship. 1 2 3 4

C17 I sometimes wish I had not married my presents spouse. 1 2 3 4

C18 My spouse lacks respect for me. 1 2 3 4

C19 I have definite difficulty confiding in my spouse. 1 2 3 4

C20 I am definitely satisfied with my marriage. 1 2 3 4

**D Your opinion on the possible harm of Secondhand Smoke(SHS) in pregnant women.**

Please mark "√" on the number that match your opinion.

Strongly Generally Generally Strongly

agree agree disagree disagree

D1 Exposure to SHS can cause lung cancer. 1 2 3 4

D2 Exposure to SHS can cause heart disease. 1 2 3 4

D3 Exposure to SHS can cause stroke. 1 2 3 4

D4 Exposure to SHS can impede embryo development. 1 2 3 4

D5 Exposure to SHS can lead to spontaneous abortion. 1 2 3 4

D6 Exposure to SHS can lead to low birth weight babies. 1 2 3 4

**We would like to thank you again for your attention and participation!**
